# Placental 11-Beta Hydroxysteroid Dehydrogenase Methylation Is Associated with Newborn Growth and a Measure of Neurobehavioral Outcome

**DOI:** 10.1371/journal.pone.0033794

**Published:** 2012-03-14

**Authors:** Carmen J. Marsit, Matthew A. Maccani, James F. Padbury, Barry M. Lester

**Affiliations:** 1 Departments of Pharmacology and Toxicology and Community and Family Medicine Section Epidemiology and Biostatistics, Dartmouth Medical School, Hanover, New Hampshire, United States of America; 2 Division of Behavioral Genetics, Rhode Island Hospital, Providence, Rhode Island, United States of America; 3 Center for Alcohol and Addiction Studies, Brown University, Providence, Rhode Island, United States of America; 4 Department of Pediatrics, Women & Infants Hospital of Rhode Island, Providence, Rhode Island, United States of America; 5 The Brown Center for the Study of Children at Risk, Women & Infants Hospital of Rhode Island, Providence, Rhode Island, United States of America; 6 Brown Alpert Medical School, Providence, Rhode Island, United States of America; VU University Medical Center, The Netherlands

## Abstract

**Background:**

There is growing evidence that the intrauterine environment can impact the neurodevelopment of the fetus through alterations in the functional epigenome of the placenta. In the placenta, the *HSD11B2* gene encoding the 11-beta hydroxysteroid dehydrogenase enzyme, which is responsible for the inactivation of maternal cortisol, is regulated by DNA methylation, and has been shown to be susceptible to stressors from the maternal environment.

**Methodology/Principal Findings:**

We examined the association between DNA methylation of the *HSD11B2* promoter region in the placenta of 185 healthy newborn infants and infant and maternal characteristics, as well as the association between this epigenetic variability and newborn neurobehavioral outcome assessed with the NICU Network Neurobehavioral Scales. Controlling for confounders, *HSD11B2* methylation extent is greatest in infants with the lowest birthweights (P = 0.04), and this increasing methylation was associated with reduced scores of quality of movement (P = 0.04).

**Conclusions/Significance:**

These results suggest that factors in the intrauterine environment which contribute to birth outcome may be associated with placental methylation of the *HSD11B2* gene and that this epigenetic alteration is in turn associated with a prospectively predictive early neurobehavioral outcome, suggesting in some part a mechanism for the developmental origins of infant neurological health.

## Introduction

The period of intrauterine development is recognized as a critical period during which the environment experienced by the mother and developing fetus can have profound effects on the later health of the infant. While the epidemiologic data is clear, the molecular basis of these effects remain to be elucidated, although epigenetic regulation likely plays a central role as a mediator of this fetal programming of adult health. These mechanisms, which include processes such as DNA methylation and post-translational modification of histones can be passed on through cell division and are thus considered stable mechanisms of maintaining cellular control, in a tissue-specific pattern, throughout life. Evidence is accumulating that these epigenetic mechanisms are susceptible to environmental signals, and can be altered, particularly at critical periods of development [1].

Much of the literature on the developmental origins of health and disease has focused on the relationship between growth, as reflected by infant birth weight, and its association with cardiovascular and metabolic outcomes [2], [3], [4]. Yet, the effects of epigenetic changes are not limited to metabolic outcomes, and infant growth has now been linked to various neurodevelopmental and mental health outcomes including schizophrenia [5], depression [6], [7], [8], and psychological distress [9], [10], [11]. Birth weight itself does not likely lead directly to these outcomes, but instead represents an integrated reflection of the in utero period and the environment experienced by the developing fetus [12].

The environment experienced by the fetus is regulated by the placenta, which plays an active immune-endocrine functional role in pregnancy, in addition to its role in nutrient, gas, and waste exchange. Rapid advancements in the discovery of the integrated regulation of neuropeptide homeostasis within and outside the brain as well as placenta [13], [14], [15] has led to the formulation of a new concept that the placenta is the “third brain” that links the developed (maternal) and developing (fetal) brains [14]. We have expanded this concept to understanding of the pathophysiology of intrauterine insults [16]. Specifically, the placenta plays a key role in the appropriate development of the HPA axis, and we have shown that stress factors during pregnancy, resulting from various adverse intrauterine environments, are associated with epigenetic alterations leading to reduced placental 11beta-hydroxysteroid dehydrogenase type 2 (*HSD11B2*) and norepinephrine transporter (*NET*) gene expression resulting in increased circulating catecholamine levels in the fetus and placental microenvironment, increased fetal exposure to cortisol, and dysregulation of the infant's HPA axis and neurobehavior [16], [17]. In this study, we are specifically asking how measures of infant growth are associated with functional epigenetic alterations in the placental *HSD11B2* gene promoter, and if epigenetic alterations to this gene are associated with prospective, validated neurobehavioral profiles.

## Results

The demographics of the 186 infants in our population that were studied are provided in Table 1. Based on the intended composition of the cohort, the population is over-represented by small and large for gestational age infants (SGA and LGA, respectively). All infants enrolled were considered at or near term, with a mean gestational age of 39.1 weeks. The racial profile of our population is mixed, with 10% of mothers reporting an African-American race, and over 20% a race other than caucasian or African American. Only 4% of participants reported smoking tobacco during pregnancy, and less than 1% reported alcohol or illegal drug use during pregnancy. The sociodemographic characteristics of the population is also mixed, with 54% of the population utilizing public health insurance. Also included in Table 1 are the descriptive statistics of the summary scores for the 10 NNNS measures examined in this study.

**Table 1:** Population Demographics, Clinical Characteristics, and NNNS Summary Scores.

	N	%	Mean	Std. Dev.	Median	Min	Max
**Categorical Variables**							
Growth Status							
SGA	53	28					
AGA	100	54					
LGA	33	18					
Infant Gender							
Female	101	55					
Male	84	45					
Maternal Race							
Caucasian	124	67					
African American	19	10					
Other	39	21					
Unknown	4	2					
Maternal Insurance							
Public	101	54					
Private	85	46					
Maternal Tobacco Use During Pregnancy							
No	176	95					
Yes	8	4					
Unknown	2	1					
**Continuous Variables**							
Birth weight (g)	186		3240.8	685.1	3132.5	1705	4730
Gestational Age (weeks)	186		39.1	1.1	39.2	37.0	41.2
Maternal Age (yrs)	186		28.3	5.8	29.0	18.0	40.0
Habituation	98		7.0	1.5	7.3	1.0	9.0
Attention	160		4.1	1.2	4.1	1.6	7.6
Stress Abstinence	186		0.21	0.07	0.20	0.06	0.41
Quality of Movement	186		4.0	0.69	4.1	2.2	5.5
Excitability	186		4.7	3.0	5	0	13
Handling	186		0.37	0.24	0.38	0.0	1.0
Self-regulation	185		4.7	0.9	4.6	2.3	7.0
Arousal	186		4.1	0.9	4.1	1.9	6.3
Hypertonicity	186		0.33	0.73	0	0	5
Hypotonicity	186		0.61	0.98	0	0	7
Asymmetrical Reflexes	186		2.0	1.4	2	0	7
Lethargy	186		6.2	2.7	6	1	14
Non-optimal Reflexes	186		6.4	2.2	7	0	11

Note: Not all N values equal 186 because of missing values.

**Figure 1:**
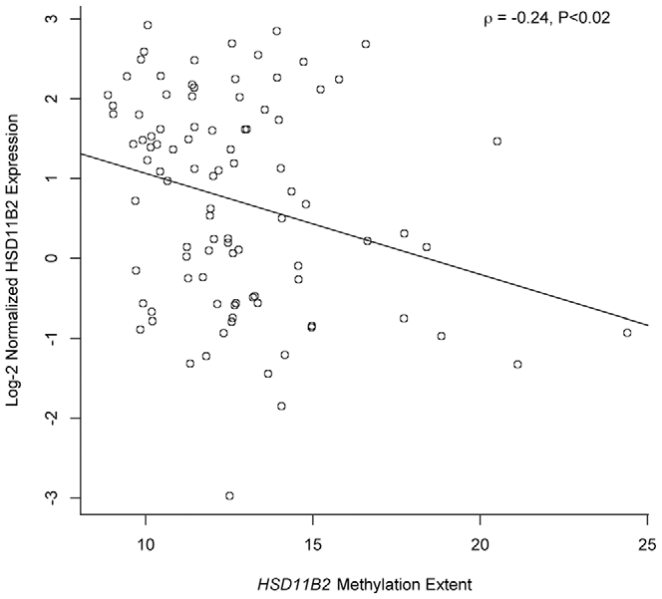
Scatterplot of the correlation between mean *HSD11B2* methylation extent and normalized HSD11B2 mRNA expression. The Spearman correlation coefficient (ρ) and associated P-value provided.

**Figure 2:**
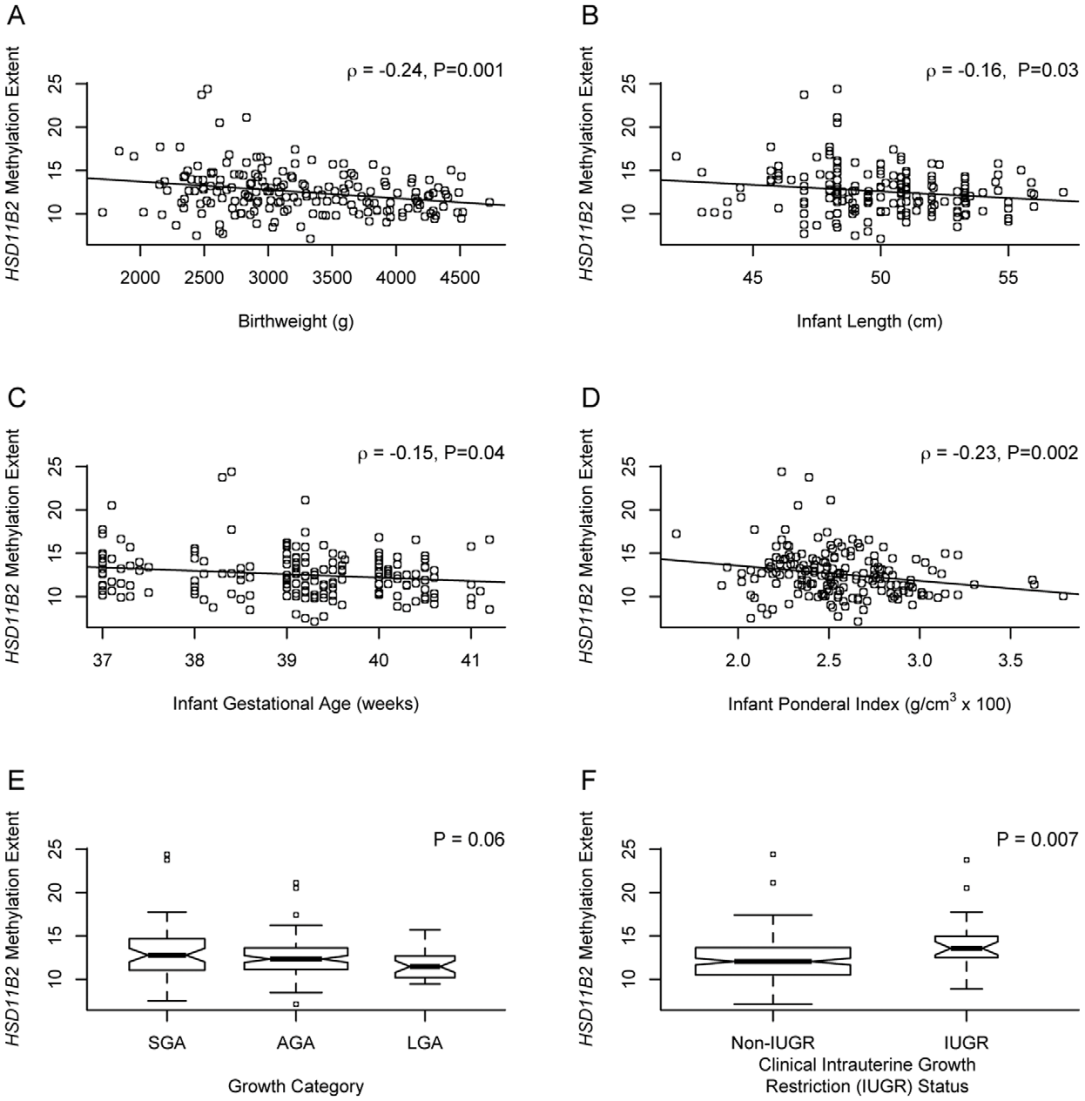
Plots of the correlations between *HSD11B2* mean methylation extent and infant characteristics. Scatterplots depict the correlation of *HSD11B2* mean methylation extent (y-axis) and (A) infant birthweight in g, (B) infant length in cm, (C) infant gestational age in weeks, and (D) infant ponderal index in g/cm^3^×100 on the x-axes, with the Spearman correlation coefficient (ρ) and associated P-value provided. Boxplots representing *HSD11B2* mean methylation extent (y-axis) by (E) growth category (small for gestational age = SGA, appropriate for gestational age = AGA, large for gestational age = LGA) and (F) clinical intrauterine growth restriction (IUGR) diagnosis (x-axes). Width of box is proportional to square root of n within each group, with the median depicted as the thick horizontal line centered in the box, the 25^th^ and 75^th^ percentiles as the outer edges of the box, and the 5^th^ and 95^th^ percentiles the whiskers. P-values represent the results of a non-parametric Kruskal-Wallis Test (E) and Mann-Whitney U-test (F).

Quantitative bisulfite pyrosequencing was used to determine the DNA methylation status of a CpG island region in the promoter of the *HSD11B2* gene previously shown to exhibit differential methylation in human placenta tissue in our sample of 186 placentas [18]. To determine the functional significance of the variation in methylation in these samples, we also performed gene expression analysis using qRT-PCR in a subset of 95 placentas examined. A moderate, statistically significant negative correlation (ρ = −0.24, P<0.02) was observed between mean extent of *HSD11B2* methylation across the 4 CpG loci examined and mRNA expression of *HSD11B2* (Figure 1). We have previously demonstrated a relationship between infant birth weight status and DNA methylation of the glucocorticoid receptor promoter [19] and so examined the association between various measures of infant growth and maternal and infant clinical characteristics and overall mean methylation of the *HSD11B2* gene promoter region. Figure 2 presents the bivariate correlations between mean extent of *HSD11B2* methylation and each of these characteristics. Moderate, statistically significant negative, correlations were observed between infant birthweight, and ponderal index (ratio of weight for length) and *HSD11B2* methylation, demonstrating smaller and leaner/thinner infants had increased levels of *HSD11B2* methylation. This was clearly shown by comparison of the extent of *HSD11B2* methylation in intrauterine growth restricted vs. non-IUGR infants, where IUGR infants demonstrated a significantly greater extent of *HSD11B2* methylation (P = 0.007, Figure 2F). More modest, yet statistically significant negative correlations were observed between HSD11B2 methylation extent and infant length and gestational age.

The relationship between these measures of growth and *HSD11B2* methylation extent, controlled for infant gender, maternal age, and gestational age are provided in Table 2. There is a consistent negative association between birthweight, ponderal index and gestational age and the extent of methylation in the *HSD11B2* promoter, but the association between infant length and methylation is attenuated in these adjusted models. The relationship is particularly strong, consistent with the bivariate tests, between IUGR status and methylation. Taken together, these results demonstrate that specific measures of intrauterine growth are associated with DNA methylation of this gene in the placenta.

**Table 2:** Generalized Linear Models of the Association between HSD11B2 Methylation Extent and Maternal and Infant Characteristics Controlled for Confounders.

Characteristics	Estimate (Standard Error)	*P*
**Infant Birthweight (per kg)** a	−0.023	0.04
**Infant Gestational Age (per week)** b	−0.014	0.01
**Infant Ponderal Index (per g/cm^3^×100)** a	−0.04	0.03
**Infant Length (per cm)** c	0.005	0.2
**Growth Category** a		
**Small for Gestational Age**	0.01	0.3
**Appropriate for Gestational Age**	Referent	
**Large for Gestational Age**	−0.02	0.2
**Clinically Diagnosed Intrauterine Growth Restriction** a		
**Non-IUGR**	Referent	
**IUGR**	0.04	0.04

Note: Each characteristic was modeled as the independent variable associated with log-transformed HSD11B2 Methylation extent as the dependent variable. Models controlled for

a infant gestational age, gender, and maternal age;

b gender and maternal age;

c birthweight, gestational age, gender, and maternal age.

**Figure 3:**
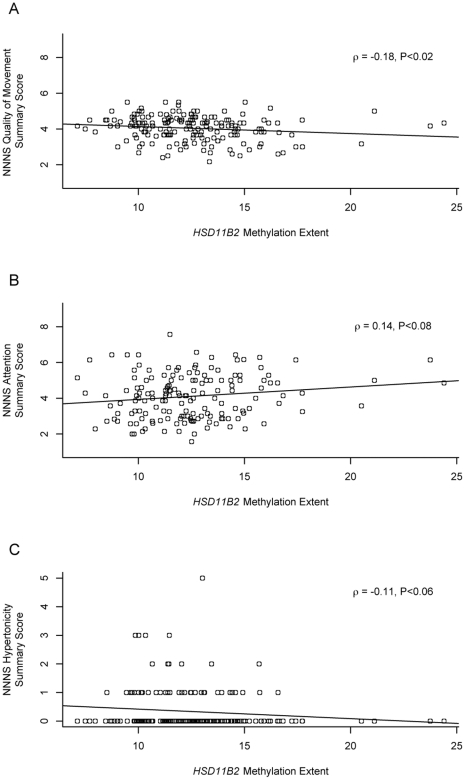
Plots of the relationships of *HSD11B2* mean methylation and selected NICU Network Neurobehavioral Scales. Scatterplots of the correlations between *HSD11B2* mean methylation extent (x-axis) and infant NICU Network Neurobehavioral Scales (A) Quality of Movement, (B) Attention, and (C) Hypertonicity scores (y-axes). Values of the Spearman correlation coefficient (ρ ) and its P-value are presented with each plot.

The relationship between mean methylation extent of the *HSD11B2* gene promoter and each of the individual NNNS summary scores was examined using a Spearman rank correlation, the results of which are tabulated in Table 3. Infant quality of movement scores showed a significant negative correlation, suggesting that increased *HSD11B2* methylation was associated with poorer quality of movement (ρ = −0.18, P = 0.01). Infant hypertonicity also showed a negative trend in its association with HSD11B2 methylation (ρ = −0.11, P = 0.06), while infant attention showed a positive trend in its association with *HSD11B2* methylation, with increasing methylation related to increasing infant attention scores (ρ = 0.14, P = 0.08). Figure 3 depicts these relationships. The other scores were not associated with *HSD11B2* methylation at the P<0.1 level.

**Table 3:** Bivariate Correlations Between HSD11B2 Mean Methylation Extent and NICU Network Neurobehavioral Scales Summary Scores.

NNNS Summary Score	N	Spearman Correlation Coefficient with HSD11B2 Methylation	*P*
**Habituation**	97	0.11	0.3
**Attention**	159	0.14	0.08
**Stress Abstinence**	185	0.06	0.4
**Quality of Movement**	185	−0.18	0.01
**Excitability**	185	0.02	0.8
**Handling**	185	−0.05	0.5
**Self-regulation**	184	−0.01	0.9
**Arousal**	185	−0.02	0.8
**Hypertonicity**	185	−0.11	0.06
**Hypotonicity**	185	−0.06	0.4
**Asymmetrical Reflexes**	185	−0.07	0.3
**Lethargy**	185	−0.06	0.5
**Non-optimal Reflexes**	185	−0.02	0.7

To examine if the association between NNNS outcomes and *HSD11B2* methylation was independent of infant growth, gender, and maternal age, we utilized multivariable generalized linear regression to model NNNS outcomes (quality of movement, hypertonicity, and attention) with *HSD11B2* methylation alone, or controlling for these additional factors. The results of these models including the effect sizes (ß values) and statistical significance are shown in Table 4. As expected from the univariate correlation analyses, in the unadjusted model, an increase in methylation of 10 fold (i.e. 1 log) of the *HSD11B2* promoter region was significantly associated with a decrease in infant quality of movement of 1.3 units. Controlling for infant growth, gender, and maternal age attenuated slightly the effect, of *HSD11B2* methylation on quality of movement, with a 10-fold increase of methylation now associated with a significant decrease in quality of movement of 1.2 units, independent of growth, gender and maternal age. Interestingly, each year of maternal age was associated with a small but significant increase in quality of movement independent of the other factors. On the other hand, a 10-fold increase in methylation showed a trend for an increase in infant attention score of 1.89 units, which was attenuated and not significant when controlled for infant growth, as well as infant gender and maternal age. Hypertonicity decreased by nearly 1 unit with a 10-fold increase in HSD11B2 methylation, a result that also was attenuated and not significant when confounders were included in the model.

**Table 4:** Generalized Linear Models of the Association between HSD11B2 Methylation Extent and NICU Network Neurobehavioral Scales Quality of Movement, Attention, and Hypertonicity Scales.

	Unadjusted		Adjusted	
	Estimate (Standard Error)	P	Estimate (Standard Error)	P
**Quality of Movement**				
**Log HSD11B2 Mean Methylation Extent**	−1.29 (0.58)	0.03	−1.18 (0.58)	0.04
**Infant Growth Status**				
Small for Gestational Age	–		−0.08 (0.12)	0.5
Appropriate for Gestational Age	–		Referent	
Large for Gestational Age	–		0.04 (0.14)	0.8
**Infant Gender**				
Female	–		Referent	
Male	–		0.03 (0.10)	0.7
**Maternal Age (yrs)**	–		0.02 (0.01)	0.01
**Attention**				
**Log HSD11B2 Mean Methylation Extent**	1.89 (1.1)	0.09	1.56 (1.13)	0.17
**Infant Growth Status**				
Small for Gestational Age	–		−0.15 (0.23)	0.5
Appropriate for Gestational Age	–		Referent	0.2
Large for Gestational Age	–		−0.36 (0.28)	
**Infant Gender**				
Female	–		Referent	
Male	–		−0.24 (0.20)	0.2
**Maternal Age (yrs)**	–		−0.002 (0.02)	0.9
**Hypertonicity**				
**Log HSD11B2 Mean Methylation Extent**	−0.92 (0.61)	0.13	−0.69 (0.61)	0.3
**Infant Growth Status**				
Small for Gestational Age	–		0.06 (0.12)	0.6
Appropriate for Gestational Age	–		Referent	
Large for Gestational Age	–		0.51 (0.14)	0.0005
**Infant Gender**				
Female	–		Referent	
Male	–		−0.07 (0.11)	0.5
**Maternal Age (yrs)**	–		−0.010 (0.010)	0.3

## Discussion

Alteration to the placental expression of genes involved in the HPA axis, including those involved in cortisol metabolism have been linked to stresses during the intrauterine period and to adverse pregnancy outcomes [16], [17], [20], [21]. This study was aimed at addressing if and how epigenetic regulation of the *HSD11B2* gene, a key gene involved in cortisol regulation in the placenta, is related to infant growth and to prospectively validated neurobehavioral outcomes in newborns. We have demonstrated an inverse association between measures of intrauterine growth, including birth weight and ponderal index with the extent of DNA methylation of the *HSD11B2* gene promoter. Growth restricted infants, and particularly those with clinically diagnosed intrauterine growth restriction, demonstrate significantly greater methylation than their appropriately grown counterparts. We also identified a significant inverse relationship between the extent of *HSD11B2* methylation and infant quality of movement. There was also a trend toward a positive correlation between *HSD11B2* methylation and infant attention.

In both animal models and human studies, maternal stress, defined broadly to include illicit drug exposures, tobacco smoking, anxiety, and depression, and their accompanying physiologic responses has been linked to the downregulation of placental *HSD11B2* gene expression, and accompanying increased fetal exposure to cortisol [16], [17], [22], [23], [24], [25]. In turn, this downregulation of HSD11B2 has been linked to growth restriction in rats [26], and humans [27], [28]. We have previously observed that the glucocorticoid receptor also undergoes functional DNA methylation resulting in reduced expression in the placenta, but found the lowest levels of methylation of this receptor in SGA infants [19]. This might suggest that an adverse intrauterine environment leading to growth restriction may enhance infant cortisol exposure and its downstream effects both by reducing HSD11B2 expression and by allowing glucocorticoid receptor expression by maintaining low levels of methylation at that promoter. The enhanced levels of active cortisol and potentially enhanced response may then be responsible for inappropriate programming of the HPA axis [29], [30] as well as altered neuromuscular development in the infant [31], [32], demonstrated by our findings with infant quality of movement.

Animal models have also linked reduced *HSD11B2* gene expression to adverse neurobehavioral outcomes including increased anxiety and stress in adult rodents [33], [34], [35]. In humans, increased cortisol levels at particular periods of pregnancy, in a sexually dimorphic manner, have been linked to reduced infant neuromuscular maturation [31]. Consistent with the concept of “critical developmental periods,” neonatal treatment of premature infants with exogenous glucocorticoids has recently been linked to adverse neurodevelopmental outcomes [36] while this effect may be confounded by many additional factors [37]. Our data suggest that epigenetic regulation of *HSD11B2* gene expression is associated with alterations of the maternal environment which affect intrauterine growth. These alterations in turn, play a role in neurobehavioral development, particularly with neuromuscular development measured as quality of movement. This measure has been shown to predict poorer performance on the 24 month Bayley Psychomotor Developmental Index and cerebral palsy at 2 years [38], [39], as well a with school readiness and behavior problems and IQ at age 4 to 4.5 years [40], suggesting potential predictive value of these measures and DNA methylation of *HSD11B2*.

Though not statistically significant, it is of interest that *HSD11B2* methylation correlated positively with infant attention scores. Our sample size was smaller for this component, as calculation of the attention summary score requires a minimum number of items, which may not be collected during every assessment [41]. Nonetheless, this observation, may suggest that reduced expression of *HSD11B2*, and subsequently fetal exposure to maternal cortisol may condition appropriate neurodevelopment. Alternatively, enhanced infant attention may predict an inability to block out stimulation and thus placing these children at risk for over-stimulation and distraction [42]. Further studies using larger populations are needed to better model what may be a complex relationship between these factors.

This study is unique in its focus on a healthy, near term population of infants from uncomplicated pregnancies. The hypothesis, that the placenta plays a critical role in determining downstream neurobehavioral is novel. This study represents one of the first to link growth, epigenetic alterations of placental genes, and early life neurobehavioral outcomes in a human population. Our findings associate growth to epigenetic alterations of the *HSD11B2* gene, which is central to HPA axis development, and in turn correlate these epigenetic alterations to altered infant neurobehavioral development suggesting this is a critical pathway for fetal programming and may play a role in neurobehavioral health throughout life. Like all human studies, we are limited in our ability to conclusively define the mechanism linking the intrauterine environment to epigenetic modulation and in turn infant quality of movement, but these studies provide impetus for further work in model systems where such mechanisms can be ascertained using controlled studies. Along those lines, we also cannot demonstrate the causality of growth on methylation of HSD11B2 and its effect on infant quality of movement, and our power is limited to examine how growth may be altering the association between HSD11B2 methylation and infant quality of movement. Expansion of these studies with larger samples sizes would be necessary to identify this effect modification. Future studies examining additional genes and pathways central to these outcomes are also needed and can provide novel markers for risk thereby allowing for earlier intervention and more successful treatment.

## Methods

### Study Population

Study subjects are part of the ongoing Rhode Island Child Health Study, which enrolls mother-infant pairs following delivery at Women and Infants Hospital of Rhode Island. Term infants born small for gestational age (SGA, lowest 10^th^ percentile), or large for gestational age (LGA, highest 10^th^ percentile), based on birthweight and gestational age and calculated from the Fenton growth chart [43], are selected; infants appropriate for gestational age (AGA) matched on gender, gestational age (±3 days), and maternal age (±2 years) are also enrolled. Only singleton, viable infants are included in the study. Other exclusion criteria are maternal age<18 years or a life-threatening medical complication of the mother, and congenital or chromosomal abnormality of the infant. A structured chart review was used to collect information from the maternal inpatient medical record from delivery, and mothers were subjected to an interviewer-administered structured questionnaire to obtain information on the lifestyle, demographics, and exposure histories of the participants. The NICU Network Neurobehavioral Scales (NNNS) examination was administered during the newborn inpatient stay, prior to discharge by certified psychometrists, who were blinded to the study hypothesis. The NNNS is a comprehensive evaluation of the neurobehavioral performance of high-risk term and preterm infants, that includes neurological and behavioral measures and signs of stress [44]. Items for the NNNS were scored by using previously established summary scores [40]. For this analysis, 185 subjects, the first participants with NNNS data enrolled between September 2009 and September 2010, were examined. All subjects provided written informed consent for participation under appropriate protocols approved by the Institutional Review Boards for Women and Infants' Hospital and Brown University.

### Placenta Sample Collection, Nucleic Acid Extraction and Bisulfite Modification

For each subject and within 2 hours of delivery, 12 samples of placenta tissue, 3 from each of 4 quadrants (totaling approximately 1 g of tissue) were excised. All samples were taken from the maternal side of the placenta, 2 cm from the umbilical cord insertion site, free of maternal decidua. The samples were placed immediately in RNAlater and stored at 4°C. At least 72 hours later, placenta samples were removed from RNAlater, blotted dry, snap-frozen in liquid nitrogen, homogenized using a mortar and pestle, and stored in sample tubes at −80°C until needed for examination. DNA was extracted from the placenta samples using the QIAmp DNA Mini Kit (Qiagen, Inc.), and RNA was extracted using the RNeasy mini kit (Qiagen) following manufacturer's protocols. Purified DNA and RNA were quantified using a ND-1000 spectrophotometer (Nanodrop, Wilmington, DE), and DNA samples (1 µg) were bisulfite-modified using the EZ DNA Methylation Kit (Zymo Research, CA, USA.) and stored at −20°C. RNA samples were aliquoted and stored at −80°C and samples were thawed only once for expression analysis.

### Bisulfite pyrosequencing DNA methylation analysis

Pyrosequencing was performed on PCR product amplified from bisulfite-modified DNA as described previously based on the region sequenced and displaying differential methylation in human placenta from Alikhani-Koopaei et. al [18]. In brief, HotStar Taq DNA Polymerase (Qiagen) and the following forward and biotinylated reverse primers were used for amplification: HSD11B2-F, 5′-GGAAGTGGGGTTGTGYGTTTTTAGGTTTAAGTT -3′ and HSD11B2-R, 5′-biotin-ATACCCTTTACTAATCRCACCACC-3′ (IDT Inc., Coralville, IA). Cycling conditions were 94°C for 15 minutes followed by 45 cycles of 94°C for 30 seconds, 55°C for 1 minute and 72°C for 1 minute with a final extension of 7 minutes at 72°C. PCR products were sequenced using a PyroMark MD system and the following sequencing primer (IDT): HSD11B2-seq, 5′-GGGGTAGAGATTTTAAGAA -3′. The sequencing primer was designed to sequence four CpG sites, and the dispensation orders for the assays was GTCGATGTCAGTCGTTAGTTCGTA. The percent methylation at each CpG site was quantified using the Pyro Q-CpG software, version 1.0.9. (Qiagen). In order for a sample's methylation extent to be called, it must exhibit at least a 93% bisulfite conversion rate, as assessed by pyrosequencing, and all samples examined exhibited a rate >95%. To prevent batch effects from bisulfite treatments interfering with the analysis, samples were randomized across batches.

### Gene Expression

Expression of the HSD11B2 mRNA was measured using commercially available Taqman Gene Expression Assays (Applied Biosystems, Valencia, CA) on an Applied Biosystems 7500 Real Time PCR system and analyzed with 7500 System Software. All reactions were run in triplicate, with GAPDH serving as a referent. A pooled sample of RNAs from placentas not used in this study was run on each plate and served as a reference sample to allow normalization using the ΔΔC_t_ method.

### Statistical Analysis

The mean extent of methylation across all 4 CpG sites was utilized in all analyses. Correlation between mean methylation and the log-transformed normalized gene expression was assessed using non-parametric Spearman rank correlation analysis. As the extent of methylation was skewed and not normally distributed, bivariate associations between continuous infant and maternal characteristics or NICU Network Neurobehavioral Scales Summary Scores and log-transformed methylation were assessed using a Spearman correlation, while bivariate associations between categorical characteristics and log-transformed methylation extent were examined using nonparametric Mann-Whitney U or Kruskal-Wallis Tests, as appropriate. Generalized linear models were used to model associations between methylation extent and infant characteristics or NICU Network Neurobehavioral Scales Summary Scores controlled for confounders. To satisfy the underlying assumptions of a linear model, a log_10_ transformation was applied to methylation extent in order to approximate a normal distribution. Final models of infant quality of movement, attention scores, and hypertonicity and HSD11B2 methylation included infant growth status (SGA, AGA, LGA), infant gender, and maternal age. We also examined models including maternal tobacco use and insurance as a marker of socioeconomic status, but these factors showed no significant association and did not alter the effect estimates of the other factors, and so were not considered confounders and for parsimony were removed from the models. Data were analyzed in R.
